# Efficiency of Alginic Acid, Sodium Carboxymethylcellulose, and Potassium Polyaspartate as Calcium Tartrate Stabilizers in Wines

**DOI:** 10.3390/foods13121880

**Published:** 2024-06-15

**Authors:** Fernanda Cosme, Luís Filipe-Ribeiro, Ana Coixão, Mário Bezerra, Fernando M. Nunes

**Affiliations:** Chemistry Research Centre-Vila Real (CQ-VR), Food and Wine Chemistry Laboratory, University of Trás-os-Montes and Alto Douro, 5000-801 Vila Real, Portugal; fcosme@utad.pt (F.C.); fmota@utad.pt (L.F.-R.); ana_coixao@hotmail.com (A.C.); mariojbezerra02@gmail.com (M.B.)

**Keywords:** wine, calcium, tartaric instability, calcium tartrate instability, alginic acid, sodium carboxymethylcellulose, potassium polyaspartate

## Abstract

The instability of calcium tartrate (CaT) in wines occurs when the effective concentration of ions surpasses the solubility product, leading to the formation of CaT crystals. Unlike potassium hydrogen tartrate (KHT), temperature has little effect on the rate of CaT precipitation, making cold stabilization ineffective. Additives like metatartaric acid and carboxymethylcellulose (CMC) have been used to mitigate this problem, but metatartaric acid’s effectiveness is limited due to hydrolysis. Additionally, potassium polyaspartate (KPA), commonly used as a KHT stabilizer, has been reported to reduce wine stability regarding CaT instability. Therefore, exploring alternative stabilization methods is crucial. Alginic acid, permitted as a processing aid in winemaking, can be an alternative to CMC and metatartaric acid due to its strong negative charge and ability to bind calcium ions. This study aimed to assess alginic acid’s efficacy as a CaT stabilizer compared to CMC and investigate the impact of KPA on CaT instability. The results showed that KPA did not increase CaT instability and even improved its stability in some wines. Alginic acid outperformed both CMC and KPA in mitigating CaT instability, possibly due to its higher zeta potential and calcium ion complexation ability. This study is the first to investigate the use of alginic acid for CaT stability in wine.

## 1. Introduction

Calcium is present in wine at levels ranging from 30 to 200 mg/L [1,2]. Its concentration depends not only on the amount naturally present in grapes and other parts of the bunch (endogenous origin) but also on technological operations (exogenous origin). These operations include fining with calcium bentonite, which can increase calcium content. Sodium bentonite can also increase calcium content, but to a lesser extent [3]. Other factors contributing to calcium levels in wines include deacidification with calcium carbonate [4] and storage in poorly coated cement tanks [5]. Wines with calcium levels above 70–80 mg/L are considered at risk for calcium tartrate instability [4]. This instability results in the formation of crystalline calcium L-tartrate, which appears as colorless or white, bipyramidal or rhomboid crystal deposits. In some cases, co-deposits such as phenolic and protein material, quercetin crystals, or yeast cells may also be present [6].

Calcium-induced instabilities are among the main problems encountered in bottled wines, and the precipitation of calcium tartrate (CaT) is becoming increasingly common due to the increase in calcium levels in grape must caused by climate change. Although CaT is an insoluble salt, CaT-induced instability, while less frequent than that caused by KHT, is more difficult to control and predict, as it is a slow phenomenon [7]. The time required for the spontaneous nucleation of CaT is much longer than for that of KHT [7], resulting in slower precipitation [8]. Usually, CaT precipitation occurs after aging for several years and almost always after bottling [8,9,10,11]. In addition to higher calcium levels, an increase in pH results in an increase in tartaric acid in the form of tartrate ions (T^2−^). According to Abguéguen and Boulton [7], polyphenols, proteins, and polysaccharides can act as inhibitors of CaT precipitation by interfering with the energy barrier for crystal growth. Tannins, proteins, and high-molecular-weight compounds can delay or even inhibit nucleation by binding to calcium and/or tartrate ions, thereby reducing their concentration in solution and decreasing the degree of supersaturation or blocking the formation of nuclei. The use of sterilizing membrane filtration systems immediately before bottling the wines and the subsequent removal of natural protective colloids can explain the delayed appearance of some calcium tartrate (CaT) in bottled wines, despite being considered stabilized during bottling [7,12]. McKinnon et al. [6] showed that the initial step in the precipitation of CaT involves the formation of a soluble species of CaT, which can aggregate and lead to nucleation. The crystallization of CaT is a phenomenon similar to that observed for KHT. However, preventing the appearance of CaT crystals in bottled wines is more challenging since CaT’s solubility is not significantly affected by low temperatures, rendering cold stabilization technologies ineffective for preventing CaT precipitation [5]. Therefore, nucleation is the limiting step in CaT crystallization in wine. Unlike KHT, the primary nucleation of CaT is not induced by lowering the temperature and increasing supersaturation [7,8]. This can be explained by the insufficient activation energy in the process to initiate crystal formation through cooling [13]. To address this, the use of CaT seed crystals is of interest since they induce nucleation, making surface integration the limiting phase of the process [7]. Crystal growth occurs as a second-order reaction and is influenced by factors such as alcohol content and ionic strength [7,14]. Several studies [7,8] have shown that increasing the ethanol concentration decreases the solubility of CaT. McKinnon et al. [14] and Cole and Boulton [15] found that an increase in ethanol content leads to a decrease in the induction time (the time associated with the beginning of the nucleation process) and an increase in the rate of CaT crystallization, which may be due to the decrease in the CaT solubility product caused by the higher ethanol concentration. Abguéguen and Boulton [7] found that a decrease in alcohol content reduces the growth rate of CaT crystals. Several studies [7,8,11,14] have demonstrated the strong dependence of CaT precipitation on pH. Solutions with higher pH tend to precipitate more CaT, increasing the probability of CaT instability. This can occur, for example, after malolactic fermentation, which raises the wine’s pH.

Currently, the stability of calcium tartrate is often estimated based on the calcium concentration found in wine. Many publications indicate 80 mg/L for white and rosé wines and 60 mg/L for red wines as threshold values above which wine is considered unstable, but these values should be considered with some reservations [5]. For example, red wine with a calcium content of 60 mg/L and a pH of less than 3.5 does not produce any precipitate, whereas at a pH of 3.7 or higher, it is very likely to form abundant sediments of crystals [5]. At pH values greater than 3, the percentage of ions in the T^2−^ form increases until reaching a maximum value at a pH of about 6.5 [16]. It is known that the stability of CaT cannot be predicted from a cold stability test and that wines subjected to CaT precipitation are almost impossible to stabilize, even if kept at low temperatures for long periods [10]. Traditionally, attempts to assess the stability of wine with respect to CaT precipitation were based on the calculation of a wine concentration product and its comparison with the solubility product [17,18]. This approach is recognized as having little value as a predictor of CaT instability [19]. One major limitation to the use of the concentration product approach relates to the method of determining the calcium concentration. Generally, the calcium concentration is measured by atomic absorption spectrophotometry, which overestimates the actual ionized calcium concentration [20]. An alternative approach for assessing the potential instability of wine with respect to calcium tartrate precipitation is based on the so-called mini-contact process [19,21]. In this procedure, the wine is stirred in the presence of CaT seed crystals while the conductivity of the solution is monitored. A decrease in conductivity indicates the loss of CaT from the wine. The procedure is time-consuming and is highly dependent on the quality of the seed crystal surface. Although KHT and CaT share similar crystallization systems, the addition of potassium bitartrate crystals does not induce calcium tartrate crystallization. However, the crystallization of CaT can induce the crystallization of KHT [22] or even facilitate the simultaneous stabilization of both KHT and CaT [13]. Therefore, the more specific method developed by Abguéguen and Boulton [7] was used. This method uses micronized calcium tartrate as a precipitation nucleus to induce the precipitation of calcium tartrate when its concentration in wines is above the saturation point. By determining the variation in wine calcium levels due to precipitation, a more specific measure of calcium tartrate instability can be obtained.

Traditionally, CaT instability in wine is addressed by removing calcium through electrodialysis [23,24] or treatment with cation-exchange resins [24,25], by adding D,L-tartaric acid to remove calcium by forming calcium D,L-tartrate [24,25], or by adding micronized calcium tartrate [24]. However, there are some limitations to these technologies, such as long processing times and expensive equipment. Enological stabilizing additives, which offer the advantages of simple operation and low cost, have been used. Stabilizing additives in wine that are allowed by the EU and OIV and are efficient for calcium tartrate instability include metatartaric acid [24] and sodium carboxymethylcellulose [24]. Nevertheless, metatartaric acid is a short-term solution because, over time, it can slowly hydrolyze into tartaric acid, which can increase the risk of precipitation [12,26,27]. These additives are also used for potassium hydrogen tartrate instability [24], but potassium polyaspartate, another widely used additive for potassium hydrogen tartrate instability, has been described as increasing calcium tartrate instability in unstable wines [28]. Therefore, evaluating alternative natural calcium tartrate stabilizers is important for expanding the range of available options for calcium tartrate stabilization in wines. Considering the chemical characteristics of alginic acid, such as its strong anionic character and affinity for calcium ions, this polysaccharide was studied for the first time to evaluate its potential to stabilize calcium tartrate. This polysaccharide is already authorized for use in the clarification of wines with no application limit [24] and is considered a processing aid by the OIV, making it a clean-label option for calcium tartrate stabilization.

## 2. Materials and Methods

### 2.1. Wine Sample

Nineteen wines from the 2021 harvest were selected, including seven white wines (WW1 to WW7), five rosé wines (RW1 to RW5), and seven red wines (REW1 to REW7). All wines came from the Vinho Verde Demarcated Region, except for WW6, which originated from the Douro Demarcated Region, and RW2, which came from the Lisbon Demarcated Region. The conventional enological parameters, including density (g/cm^3^), alcohol strength (% *v*/*v*), total acidity (g/L tartaric acid), volatile acidity (g/L acetic acid), pH, malic acid (g/L), and reducing sugars (g/L), were determined using a Bacchus micro FTIR device (Microderm, France). Free sulfur dioxide (mg/L) was determined using the Ripper method. Analyses were performed in triplicate.

### 2.2. Determination of Calcium and Potassium

Calcium and potassium were determined using flame atomic absorption spectrophotometry on an instrument equipped with an acetylene air burner and hollow cathode lamp specific to each metal being analyzed. For calcium determination, the absorbance at 422.7 nm was measured. The sample was concentrated twice, and the measurement was performed in the presence of lanthanum chloride as a “matrix modifier” [29]. To prepare the calibration curve, solutions of 2, 4, 6, and 8 mg/L calcium were prepared by taking 1.0, 2.0, 3.0, and 4.0 mL, respectively, from the 100 mg/L calcium solution. Potassium was determined by directly reading the absorbance at 766 nm. Initially, a 100 mg/L solution of potassium chloride was prepared. Then, a buffer solution was prepared by sequentially saturating 100 mL of distilled water with NaCl, CaCl_2_, and MgCl_2_, filtering the solution after each saturation. The calibration curve was constructed using 1.0, 2.0, 3.0, 4.0, and 5.0 mL of the 100 mg/L potassium solution. For each sample, 1 mL of the buffer solution was added to 100 mL of the sample, and the emission was read. All samples were analyzed in duplicate.

### 2.3. Evaluation of Wine Calcium Tartrate Stability

The stability of calcium tartrate was evaluated using the method described by Abguéguen and Boulton [7]. Initially, the calcium content of the wine was determined after centrifugation (5000 r.p.m for 10 min) and coded as C_Ca_i.

To 50 mL of wine, 0.2 g of micronized calcium tartrate (CaT) was added. The mixture was stirred for 15 min and allowed to stand at 1–2 °C for 24 h. Subsequently, the wine was filtered through a 0.45 µm membrane. The filtered wine was analyzed for calcium content and coded as C_Ca_f. If the calcium concentration increased significantly, the wine was considered stable at the test temperature, indicating that some of the added micronized calcium tartrate had dissolved. If a significant decrease in the calcium concentration was observed, the wine was considered unstable at the test temperature, indicating supersaturation and crystal growth. No changes in calcium concentration indicated that the wine was stable at the test temperature [7]. The level of wine calcium tartrate instability was determined according to Triulzi et al. [30] after calculating the decrease in calcium concentration after the application of micronized calcium tartrate, using the values presented in Table 1.

### 2.4. Stabilization Experiments Using Sodium Carboxymethylcellulose, Potassium Polyaspartate, and Alginic Acid

From the initial set of nineteen wines, nine were selected for the stabilization experiments with sodium carboxymethylcellulose, potassium polyaspartate, and alginic acid based on their level of calcium tartrate stability: three stable wines (ΔCa < 15 mg/L)—WW3, RW3, and REW6; three slightly unstable wines (15 mg/L < ΔCa < 25 mg/L)—WW1, WW2, and REW3; and three highly unstable wines (ΔCa > 25 mg/L)—RW4, REW2, and REW4. Before the addition of the tartaric stabilizers, all wines underwent a sulfur dioxide correction to 50 mg/L free sulfur dioxide using a 6% *w*/*v* sulfur solution. CMC (SAIStab CMC 10), a 10% CMC solution from SAI Enology, was added at a dose of 200 mL/hL to achieve a concentration of 200 mg/L (maximum legal dose). Zenith Uno (Enartis), a 5% potassium polyaspartate solution, was added at a dose of 100 mL/hL to achieve a concentration of 100 mg/L (maximum legal dose). ACStab alginic acid (a test product from SAI Enology) was added directly as a powder at a dose of 300 mg/L (determined in previous tests as a non-commercial product), as there is no limit for the use of alginic acid in winemaking [24]. The treatments were conducted using 180 mL of wine in each sample. The additives were thoroughly mixed with the wine and allowed to remain in contact for seven days at room temperature in duplicate samples. The same wine without additives was used as a control. After seven days’ contact time, as described above, the samples were centrifuged at 5000 r.p.m. for 10 min and tested for calcium tartrate instability (Section 2.3).

### 2.5. Zeta Potential

The charge of the polymer has a direct relationship with the zeta potential (Zp) of the solution. Zp is defined as the electrical potential that exists at the hydrodynamic plane of shear surrounding a charged particle and is essentially the potential at the point in space where low-molecular-weight ions cease to move with the particle and remain within the surrounding solvent [31]. Generally, when all the particles have a large positive or negative Zp (where the positivity or negativity is greater or lower than +30 mV or −30 mV), they will repel each other, and the dispersion is stable. On the other hand, when the particles have low Zp values, there will not be sufficient force to prevent the particles from aggregating [32]. The zeta potential was investigated by electrophoretic light scattering (ELS) (Litesizer 500, Anton Paar, Turin, Italy). The analysis was carried out at 25 °C, with 2 min of equilibration time. The Smoluchowski approximation was applied with a Henry factor of 1.50 at 200 Volts in a 10 mM NaCl solution. To avoid multiple scattering effects due to the high particle concentration, all samples were previously diluted with a 10 mM NaCl solution to a concentration of 1 mg/mL. Three replicates were performed.

### 2.6. Statistical Analysis

The Student *t*-test was employed to evaluate whether there were significant differences in each wine, both with and without additives, before and after the addition of micronized calcium tartrate. This analysis was conducted individually for each wine.

Furthermore, ANOVA was employed to assess the differences in calcium content in the calcium tartrate stability test (ΔCa) among the different treatments. Tukey’s post hoc test (HSD, 5% level) was applied to the physicochemical data to determine significant differences. Statistical significance was confirmed when *p*-values were found to be less than 0.05.

Multiple linear regression analysis (MLR) models were developed to predict the calcium tartrate instability in wines (ΔCa, dependent variable) from the chemical composition of wines, with calcium concentration (mg/L), potassium concentration (g/L), pH, total acidity (g tartaric acid/L), malic acid content (g/L), alcohol strength (% *v*/*v*), density, reducing sugars (g/L), and the concentration product of calcium and tartrate ions (T^2−^) calculated according to the wine pH as predictor variables. For model building, a forward stepwise routine with a probability of variable retention of *p* < 0.05 was performed. Multicollinearity in the data sets was assessed by determining the variance inflation factor and tolerance, and no problems were detected. Parameter estimates for all models were evaluated using *t*-tests. The model’s predictive ability was validated by leave-one-out cross-validation [33]. Data analysis was performed using Statistica 7.

## 3. Results and Discussion

### 3.1. Effect of Wine Composition on Selected Parameters for Wine Calcium Tartaric Stability

Calcium tartrate stability in nineteen wines, four white wines, three rosé wines, and two red wines (Table 2) was determined (Table 3). Calcium levels varied between 45.15 mg/L and 114.60 mg/L, in line with the normal range of calcium concentrations described for wines [1,2]. The pH range of the wines varied between 3.12 and 3.64, and in this pH range, tartaric acid exists in three forms: free acid (H_2_T), monohydrogen tartrate (HT^−^), and tartrate (T^2−^). The major form is HT^−^, with the amount of T^2−^ being only 3–9% [16].

**Figure 1:**
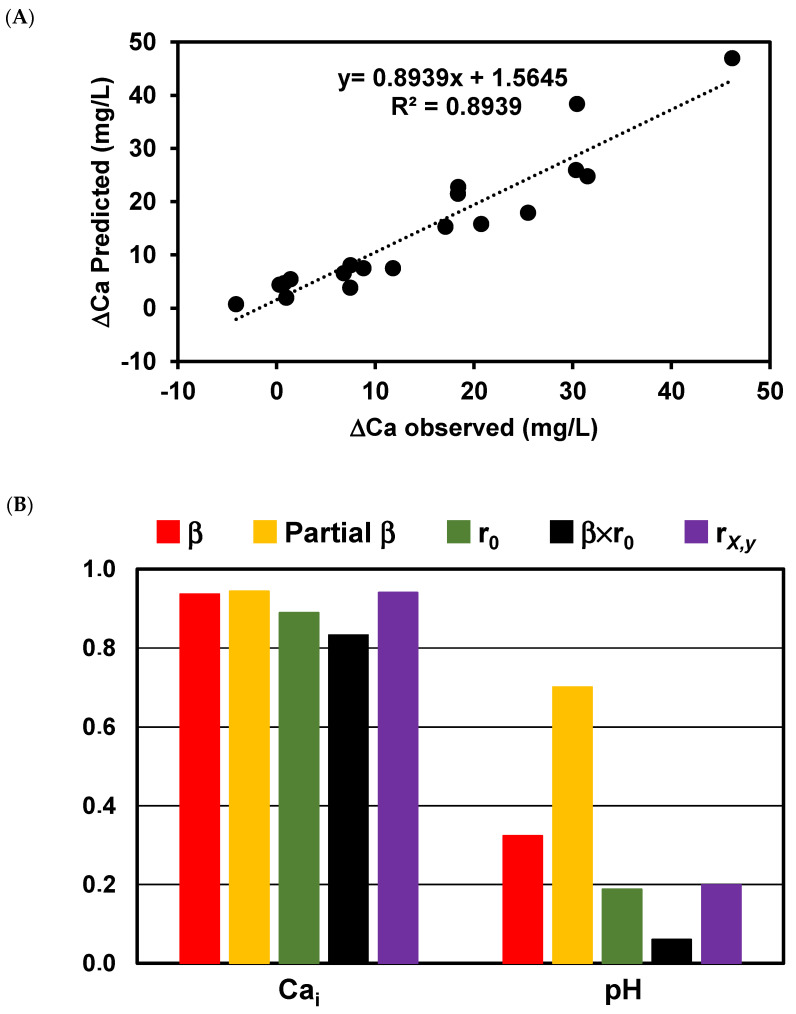
(**A**) Multiple regression analysis to predict ΔCa using Ca_i_ and pH as predictors. (**B**) The contribution of each predictor: standardized regression coefficients, semi-partial correlations, zero-order correlations, and structure coefficients of predictors for the ΔCa regression equation.

Nine of the wines analyzed were classified as stable (ΔCa < 15 mg/L): four white wines, three rosé wines, and two red wines. Additionally, four wines were categorized as slightly unstable (15 mg/L < ΔCa < 25 mg/L), including two white wines and two red wines. The remaining five showed high instability in relation to calcium tartrate (ΔCa > 25 mg/L), consisting of two rosé wines and three red wines (Table 3). For all the wine parameters analyzed, only the initial calcium concentration (R = 0.8895) and total acidity (R = 0.4885) presented a significant correlation with the calcium tartrate instability (ΔCa). Although these observed correlations were expected, it is known that other factors, such as the pH, alcohol content, organic acid content, and polysaccharides, among others, can influence calcium tartare instability [7,11,14,34,35,36]. To understand the relative contributions of the different wine variables to the measured calcium tartrate instability (ΔCa), an MLR analysis was performed using C_Ca_i, pH, alcohol strength, total acidity, C_K_i, malic acid content, density, and the concentration product as continuous predictors (Figure 1A). Multiple linear regression offers a more precise analysis compared to simple linear regression. By allowing the inclusion of multiple independent variables in one model, multiple linear regression enables us to account for various important factors simultaneously, leading to a more accurate understanding of each factor’s association with the outcome; control for confounding variables; make predictions; and identify important independent variables. Only two variables were retained in the model obtained for predicting ΔCa, namely, the initial calcium wine concentration and the wine pH. The model was statistically significant (R = 0.945; F(2,16) = 67.40; *p* < 0.0000001) and accounted for approximately 89% of the variance of ΔCa (R^2^ = 0.894, Adjusted R^2^ = 0.881) observed for the wines studied. ΔCa was primarily predicted by higher levels of calcium in the wines and, to a lesser extent, by higher pH values. This model presented a good predicting ability, as shown by the results obtained for leave-one-out cross-validation, where a root mean square error of 5.0 was obtained. The standardized regression coefficients of the predictors, together with the correlation with ΔCa, their semi-partial correlations, and their structure coefficients, are shown in Figure 1B. The wine calcium concentration obtained the largest beta weight, demonstrating that it made the largest contribution to the regression equation when holding the other predictors constant. Also, calculating the zero-order correlation coefficient (R = 0.889) shows that this variable shares a high amount of its variance with ΔCa (79%) and, to a lesser extent, the pH (4%). The squared structure correlation (rX,y) again shows that the wine calcium concentration shared the largest amount of variance (89%) with the predicted values of ΔCa, with the pH accounting only for 4%. The product measure (β × r_0_) allows the calculation of the partition of the regression effect into non-overlapping partitions based on the interaction between the beta weights of each independent variable and its zero-order correlation coefficient with the dependent variable [37], showing that, in this respect, the wine calcium concentration is responsible for much of the variation accounted for by the regression equation (83%), with less attributable to the pH (6%). These results clearly show that although, as expected, the wine calcium concentration and pH explain much of the variation observed in ΔCa, the dominant variable is the initial wine calcium concentration.

### 3.2. Effect of Stabilizing Additives on Wine Calcium Tartrate Stability

To study the effect of CMC, potassium polyaspartate, and alginic acid on the stability of calcium tartrate in wines, 9 wines with different levels of calcium tartrate instability, comprising white, rosé, and red wines, were selected from the initial screening of 19 wines described previously (Table 3). As there are some reports in the literature stating that potassium polyaspartate can induce calcium tartrate instability [28,38], three wines were chosen from each category: “stable”, “slightly unstable”, and “very unstable”. Due to the absence of significant instability in the white wines and the lack of slightly unstable rosé wines, the selection was made accordingly, as outlined in Table 4, Table 5 and Table 6. The purpose of selecting stable wines was to assess whether the addition of the stabilizers used in the treatments could potentially lead to destabilization. To investigate the impact of adding potassium polyaspartate, sodium carboxymethylcellulose, and alginic acid on the stability of calcium tartrate in wines, potassium polyaspartate and CMC were applied to the previously selected wines at their maximum allowed doses according to the OIV regulations: 100 g/hL and 200 g/hL, respectively. For alginic acid, although there is no established limit, a dose of 300 g/hL was used. The addition of potassium polyaspartate and CMC to white wines did not result in significant differences in the ΔCa of wines or decrease it significantly (Table 4). On the other hand, the addition of alginic acid always resulted in wines with significantly lower ΔCa. In fact, for the three white wines studied, two of them were stabilized by the addition of alginic acid (WW1 and WW2), and CMC and potassium polyaspartate were only able to bring wine WW2 into the stability range. Nevertheless, in no case did the addition of these additives to the white wines render the wine more unstable than the initial wines.

The same trend as that described for white wines was observed for the rosé wines after the addition of potassium polyaspartate, CMC, and alginic acid (Table 5). The addition of potassium polyaspartate and CMC resulted in no significant change or a significant decrease in ΔCa, and alginic acid consistently resulted in a significant decrease in ΔCa. For wine RW4, all additives were able to decrease the calcium instability to the stable level (Table 5).

For red wines, the application of potassium polyaspartate and CMC again resulted in no significant difference in ΔCa or a significant decrease in the ΔCa value of red wines. Alginic acid also resulted in a decrease in ΔCa; however, in red wine, this decrease was not significantly higher than that observed for potassium polyaspartate and CMC, as observed in white and rosé wines (Table 6).

Therefore, for the three-wine matrix studied, white, rosé, and red wines, with different levels of calcium tartrate instability, from stable to slightly unstable and unstable, none of the additives increased the calcium tartrate instability. Although potassium polyaspartate has been documented to potentially increase wine calcium tartrate instability, these studies were performed using much higher doses of added calcium to the wines, 400 mg/L [38] and 500 mg/L [28], and also with the addition of tartaric acid [28]. These calcium values are much higher than those described to be found in wines [1,2]. Additionally, the methods used for assessing calcium tartrate instability in these studies were either based on the mass of the precipitate formed [28] or evaluated visually for their tartrate stability and turbidity formed in the bottled wine [38]. Therefore, the nature of the precipitate was not assessed, and there is no guarantee that it consisted only of calcium tartrate crystals. As reported earlier [39,40], CMC was able to increase wine stability regarding calcium tartrate precipitation, with the exception of wines REW3 and WW1. In fact, in the present study, all additives were able to reduce the precipitation of unstable wines, although with different efficiency. This efficiency was also dependent on the wine instability, with alginic acid showing the highest stabilizing effect on calcium tartrate instability. However, this higher efficiency could also be related to the higher dose used for alginic acid [24]. CMC has been shown to be a good stabilizer for calcium tartrate instability, with the efficiency also dependent on the degree of substitution [41,42]. Therefore, the higher stabilizing effect of alginic acid may be due to its higher negative charge and its ability to complex calcium ions [43].

### 3.3. Zeta Potential of Sodium Carboxymethylcellulose, Potassium Polyaspartate, and Alginic Acid

**Figure 2:**
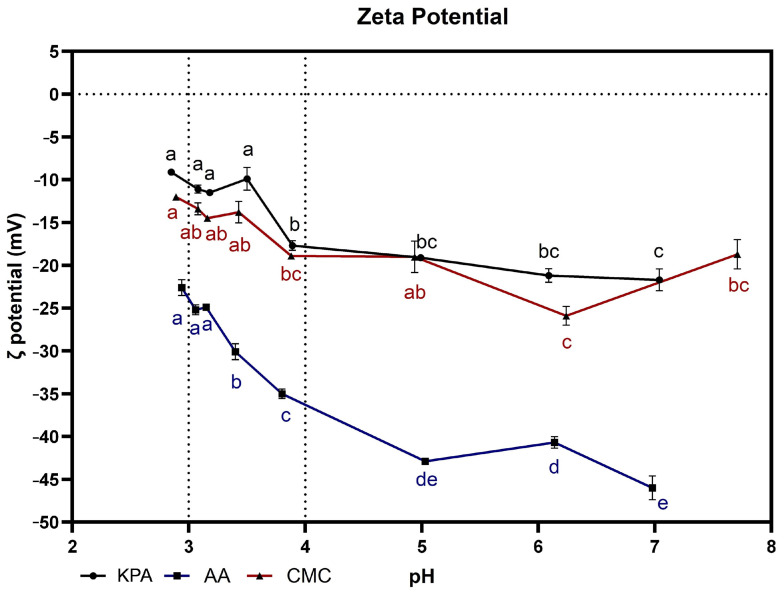
The variation in the Zp of CMC, potassium polyaspartate (KPA), and alginic acid (AA) solutions at 1 mg/mL as a function of the pH. Within each sample, values with the same letters are not significantly different after one-way ANOVA and Tukey’s post hoc test, *p* < 0.05)).

Studies carried out by McKinnon et al. [6] have shown that the first step in CaT precipitation is associated with the formation of a soluble CaT species, which, through aggregation, will result in a critical nucleus before the beginning of precipitation and crystal growth. CMC is an anionic polymer produced by the carboxymethylation of natural cellulose with sodium hydroxide and monochloroacetic acid [44]. Each of the three hydroxyl groups of the anhydrous glucose unit of cellulose can be replaced by a carboxymethyl group. Therefore, the degree of substitution (DS) of CMC can vary between 0 and 3 [45], with the reactivity of the three hydroxyl groups being OH-C2 > OH-C6 > OH-C3 [46]. CMC inhibits tartaric salt precipitation through the interaction of CMC’s negative charge with the crystals’ positive charge (potassium and calcium) [47]. Nevertheless, it has been argued that CMC also interacts with other wine compounds besides its interaction with the tartrate ions in solution, reducing the amount of K^+^ ions able to diffuse to the adsorption layer in the case of potassium tartrate precipitate. CMC, with a higher degree of substitution and lower viscosity, had the best performance in tartrate stabilization [41]. Potassium polyaspartate is a low-molecular-weight, highly polydisperse polyelectrolyte (Mn 1000 g/mol and Mw 5000 g/mol). It consists of repeat units of D- or L-potassium aspartate connected by α and β peptide bonds in a ratio of 30:70, respectively. The polypeptide is synthesized through the solid-state thermal polymerization of L-aspartic acid, resulting in polysuccinimide formation and the ring opening of the cyclic succinimide by hydrolysis with potassium hydroxide under controlled conditions [48]. Alginic acid is a linear heteropolysaccharide composed of β-D-mannuronic and α-L-guluronic acid units, which are produced by brown algae (Laminarales) [49] and some bacteria (*Azotobacter vinelandii*, *Pseudomonas aeruginosa*, and others) [50]. Alginates that are rich in guluronic acid units exhibit higher selectivity for divalent ions, such as calcium. The selectivity of alginates for divalent ions is primarily associated with the presence of guluronic acid units [51]. With one carboxyl group in each M or G unit, alginates are negatively charged polyelectrolytes as long as the pH of the solution is above the pKa of the carboxyl group (pKa ~3.0–3.5). Empirical studies have demonstrated that the electron-donating moieties of alginic acid polymers are responsible for chelating aqueous cations such as Ca^2+^ [43]. Therefore, as both potassium polyaspartate and alginic acid are negatively charged polyelectrolytes, their inhibition of calcium tartrate precipitation can follow the same mechanism as that described for CMC. The presence of the dissociated functional groups results in zeta potential changes depending on the ionic nature of polyelectrolyte; negatively charged groups (e.g., carboxylic groups) cause a decrease in ζ, whereas positively charged groups (e.g., amino groups) contribute to the increase in the zeta potential value. Therefore, the Zp values of CMC, potassium polyaspartate, and alginic acid were determined for different pH values, with a higher resolution in the pH range between 3 and 4 (Figure 2), the pH range of the wines studied (Table 2). For the same concentration, alginic acid always showed a lower Zp than CMC and potassium polyaspartate. For alginic acid, there was a significant decrease in Zp with increasing pH, even in the pH range of the wines. This is explained by the higher dissociation of the carboxylic acid groups in alginic acid with increasing pH. The same tendency was also observed for CMC and potassium polyaspartate, although the effect was less pronounced compared to alginic acid, with a significantly lower Zp observed only near pH 4 compared to pH 3. The lower Zp of alginic acid compared to CMC can be explained by the higher density of carboxylic acid groups, as all sugars present, either mannuronic or guluronic acid, contain a carboxylic acid group, while the CMC used presented a substitution degree of 0.72 [41]. Therefore, the higher charge density observed for alginic acid in the pH range studied can explain the higher stabilization effect of alginic acid on calcium tartrate precipitation when compared to CMC and potassium polyaspartate, where the two polymers showed similar behavior in the stabilization of calcium tartrate precipitation and showed similar Zp values (Figure 2).

Nevertheless, it cannot be excluded that this higher stabilizing effect of alginic acid in inhibiting calcium tartrate precipitation may also be due to the higher affinity of alginic acid for calcium ions. For example, Pellerin et al. [36] showed that among wine polysaccharides, RG-I inhibits calcium tartrate precipitation as a result of its ability to participate in calcium ion sequestration, while RG-II is much less effective. Also, arabinogalactan proteins and mannoproteins have been shown to have a reduced or even no effect on the induction time of CaT. It has been shown that alginic acid specifically interacts with the crystallographic features of calcite [52,53] and can increase the dissolution rate of calcite [53] and other minerals [54].

## 4. Conclusions

This study, to our knowledge, was the first to explore the potential use of alginic acid to stabilize wine against calcium tartrate instability and to compare its efficiency to that of CMC. When compared to CMC, alginic acid allowed for the better stabilization of wines against calcium tartare instability, resulting in a higher decrease in the precipitation of calcium tartrate and bringing most of the wines into the stability zone. This efficiency may be related to the higher concentration of alginic acid used compared to CMC (300 vs. 200 mg/L), but it is most likely due to the higher charge density of alginic acid and its ability to complex calcium ions.

Additionally, it was found that potassium polyaspartate did not increase the instability of calcium tartrate in either unstable or stable wines. In fact, it had a stabilizing performance similar to that of CMC. Although the results obtained are very promising, they only represent the short-term stabilizing effects of the three additives. Therefore, a study of the long-term stabilizing effects should be conducted with different application doses of the three additives in order to optimize their concentration and relation to the initial wine calcium tartrate instability. Also, although no negative effects are anticipated, the impact of alginic acid on the sensory characteristics of the wines should also be studied.

## Figures and Tables

**Table 1:** Reference values for defining the level of calcium tartrate instability in wines [30].

ΔCa (mg/L)	Instability Level
<15	Stable
15 ≤ ΔCa ≤ 25	Slightly unstable
>25	Very unstable

**Table 2:** The conventional enological parameters of the 19 selected wines analyzed.

	Density (20 °C) (g/cm^3^)	Alcohol Strength (% *v*/*v*)	Total Acidity (g/L Tartaric Acid)	Volatile Acidity (g/L Acetic Acid)	pH	Malic Acid (g/L)	Reducing Sugars (g/L)	Free Sulfur Dioxide (mg/L)
WHITE WINE								
WW1	0.9915	11.8	6.5	0.28	3.43	2.9	ND	28
WW2	0.9916	10.5	5.9	0.51	3.32	0	ND	6
WW3	0.9915	10.5	5.8	0.64	3.37	0	ND	6
WW4	0.9905	11.7	6.9	0.21	3.18	2.7	ND	30
WW5	0.9903	11.7	7.1	0.21	3.15	2.8	ND	13
WW6	0.9885	12.5	5.9	0.36	3.28	1	ND	33
WW7	0.9961	10.2	5.6	0.35	3.43	0.8	8.6	21
ROSÉ WINE								
RW1	0.9913	11.2	7.3	0.22	3.17	3	ND	6
RW2	0.9912	10.6	5.6	0.34	3.5	2.2	2	10
RW3	0.9916	10.4	5.4	0.36	3.32	0.9	1.4	23
RW4	0.9892	12.3	5.6	0.41	3.42	1.5	1.1	17
RW5	0.9946	10.7	5.4	0.35	3.46	1	8	15
RED WINE								
REW1	0.9972	10.4	8.7	0.39	3.31	4	ND	6
REW2	0.9974	9.9	8.7	0.46	3.27	2.9	ND	1
REW3	0.9943	9.9	7.1	0.82	3.43	0	ND	5
REW4	0.9964	9.5	7.2	0.38	3.49	0	ND	6
REW5	0.9941	10.3	7.8	0.2	3.25	3.5	ND	14
REW6	0.991	13.6	6.4	0.98	3.64	0.3	ND	15
REW7	0.9948	11.2	5.6	0.41	3.4	0	0.8	11

WW—white wine; RW—rosé wine; REW—red wine.

**Table 3:** Calcium tartrate instability in wines measured according to the method of Abguéguen and Boulton [7] and classified according to the ranges of Triulzi et al. [30].

Wine	C_Ca_i (mg/L)	C_Ca_f (mg/L)	Significance	ΔCa (mg/L)	Stability
WW1	74.65 ± 0.78	56.25 ± 2.76	*	18.39 ± 3.49	Slightly unstable
WW2	70.40 ± 1.84	49.65 ± 1.77	**	20.71 ± 0.06	Slightly unstable
WW3	56.90 ± 1.70	45.10 ± 0.00	**	11.79 ± 1.67	Stable
WW4	61.95 ± 0.50	61.15 ± 2.62	NS	0.75 ± 2.05	Stable
WW5	58.05 ± 2.90	62.15 ± 0.78	NS	−4.10 ± 2.12	Stable
WW6	56.15 ± 0.64	48.70 ± 0.85	NS	7.45 ± 1.48	Stable
WW7	51.35 ± 0.21	49.95 ± 0.64	NS	1.40 ± 0.42	Stable
RW1	66.20 ± 3.81	57.35 ± 0.78	NS	8.80 ± 4.52	Stable
RW2	74.10 ± 0.71	42.55 ± 0.35	***	31.50 ± 1.13	Very unstable
RW3	51.80 ± 1.27	50.85 ± 5.73	NS	0.99 ± 4.52	Stable
RW4	68.60 ± 2.83	43.10 ± 1.56	**	25.47 ± 1.26	Very unstable
RW5	51.50 ± 0.85	44.65 ± 0.21	**	6.80 ± 0.99	Stable
REW1	84.50 ± 3.25	54.15 ± 0.21	**	30.35 ± 3.47	Very unstable
REW2	114.60 ± 7.07	68.40 ± 4.53	*	46.17 ± 11.58	Very unstable
REW3	72.93 ± 0.11	54.57 ± 0.79	***	18.36 ± 0.90	Slightly unstable
REW4	92.84 ± 1.58	62.39 ± 0.40	**	30.44 ± 1.97	Very unstable
REW5	72.95 ± 0.07	55.80 ± 0.57	***	17.10 ± 0.71	Slightly unstable
REW6	45.15 ± 1.34	37.65 ± 1.77	*	7.49 ± 3.09	Stable
REW7	51.70 ± 1.41	51.35 ± 0.64	NS	0.30 ± 0.85	Stable

Values are presented as mean ± standard deviation (n = 2). The mean values are preceded by the significance level: *** *p* < 0.001; ** *p* < 0.01; * *p* < 0.05; NS—not significant (Student *t*-test, *p* < 0.05). C_Ca_i—initial calcium concentration; C_Ca_f—final calcium concentration. WW—white wine; RW—rosé wine; REW—red wine.

**Table 4:** Calcium tartrate instability in white wines after application of potassium polyaspartate, CMC, and alginic acid.

		C_Ca_i (mg/L)	C_Ca_f (mg/L)	Significance	ΔCa (mg/L)	Stability
WW1	T	68.23 ± 2.53	47.63 ± 0.42	**	20.60 ± 2.11 ^a^	Slightly unstable
	KPA	65.25 ± 0.84	41.07 ± 3.38	*	24.18 ± 4.22 ^a^	Slightly unstable
	CMC	66.59 ± 1.90	40.32 ± 1.48	**	26.27 ± 0.42 ^a^	Very unstable
	AA	61.81 ± 1.06	54.20 ± 3.38	NS	7.61 ± 2.32 ^b^	Stable
WW2	T	66.73 ± 4.18	49.79 ± 3.52	*	16.94 ± 7.69 ^a^	Slightly unstable
	KPA	70.92 ± 1.32	60.04 ± 0.88	*	10.88 ± 0.44 ^a^	Stable
	CMC	71.23 ± 2.20	64.55 ± 1.98	NS	6.68 ± 0.22 ^ab^	Stable
	AA	63.77 ± 3.96	71.08 ± 0.66	NS	−7.30 ± 3.30 ^b^	Stable
WW3	T	54.45 ± 0.44	43.88 ± 0.00	***	10.57 ± 0.44 ^a^	Stable
	KPA	66.26 ± 3.52	67.81 ± 0.44	NS	−1.55 ± 3.08 ^b^	Stable
	CMC	68.12 ± 2.20	64.86 ± 0.66	NS	3.26 ± 2.86 ^abc^	Stable
	AA	62.38 ± 0.66	62.06 ± 0.66	NS	0.31 ± 1.32 ^bc^	Stable

Control wine (T), wine treated with potassium polyaspartate (KPA), wine treated with sodium carboxymethyl cellulose (CMC), and wine treated with alginic acid (AA). For Ca_i_ and Ca_f_ in each wine, the mean ± standard deviation values are followed by the significance level: *** *p* < 0.001; ** *p* < 0.01; * *p* < 0.05; NS indicates the result is not significant according to the Student *t*-test, 5%. For ΔCa, within each wine, the mean ± standard deviation values following the same letter do not show statistically significant differences (ANOVA, Tukey, *p* < 0.05).

**Table 5:** Calcium tartrate instability in rosé wines after the application of potassium polyaspartate, CMC, and alginic acid.

		C_Ca_i (mg/L)	C_Ca_f (mg/L)	Significance	ΔCa (mg/L)	Stability
RW3	T	50.10 ± 1.76	51.96 ± 0.44	NS	−1.87 ± 1.32 ^a^	Stable
	KPA	50.88 ± 0.66	61.91 ± 4.39	NS	−11.03 ± 3.74 ^ab^	Stable
	CMC	48.70 ± 1.98	64.40 ± 3.08	*	−15.69 ± 1.10 ^b^	Stable
	AA	47.15 ± 3.30	61.44 ± 0.66	*	−14.30 ± 2.64 ^b^	Stable
RW4	T	66.26 ± 0.44	46.06 ± 0.00	***	20.20 ± 0.44 ^a^	Slightly unstable
	KPA	66.42 ± 0.66	57.40 ± 1.10	**	9.01 ± 1.76 ^b^	Stable
	CMC	60.98 ± 0.44	52.90 ± 1.32	*	8.08 ± 0.88 ^b^	Stable
	AA	60.36 ± 0.44	57.87 ± 0.44	NS	2.49 ± 0.88 ^c^	Stable

Control wine (T), wine treated with potassium polyaspartate (KPA), wine treated with sodium carboxymethyl cellulose (CMC), and wine treated with alginic acid (AA). For C_Ca_i and C_Ca_f in each wine, the mean ± standard deviation values are followed by the significance level: *** *p* < 0.001; ** *p* < 0.01; * *p* < 0.05; NS—not significant according to the Student *t*-test, 5%). For ΔCa, within each wine, the mean ± standard deviation values followed by the same letter do not show statistically significant differences (ANOVA, Tukey, *p* < 0.05).

**Table 6:** Calcium tartrate instability in red wines after application of potassium polyaspartate, CMC, and alginic acid.

		C_Ca_i (mg/L)	C_Ca_f (mg/L)	Significance	ΔCa (mg/L)	Stability
REW2	T	104.80 ± 6.97	65.39 ± 4.43	*	39.40 ± 2.53 ^a^	Very unstable
	KPA	93.90 ± 1.27	68.23 ± 1.69	**	25.67 ± 2.96 ^a^	Very unstable
	CMC	93.60 ± 2.96	70.17 ± 1.06	**	23.43 ± 4.01 ^a^	Slightly unstable
	AA	98.08 ± 1.69	77.78 ± 5.91	*	20.30 ± 7.60 ^a^	Slightly unstable
REW3	T	80.62 ± 0.21	54.95 ± 1.48	**	25.67 ± 1.69 ^a^	Very unstable
	KPA	81.66 ± 1.27	55.10 ± 3.38	**	26.57 ± 4.64 ^a^	Very unstable
	CMC	81.81 ± 1.90	50.17 ± 1.06	**	31.64 ± 2.96 ^a^	Very unstable
	AA	78.23 ± 1.06	56.74 ± 0.21	NS	21.49 ± 0.84 ^a^	Slightly unstable
REW4	T	57.04 ± 2.74	56.29 ± 1.69	NS	0.75 ± 1.06 ^a^	Stable
	KPA	60.02 ± 1.90	54.35 ± 1.06	NS	5.67 ± 2.96 ^a^	Stable
	CMC	59.42 ± 1.06	60.02 ± 3.17	NS	−0.60 ± 2.11 ^a^	Stable
	AA	63.45 ± 0.42	58.53 ± 3.17	NS	4.93 ± 2.74 ^a^	Stable
REW6	T	41.22 ± 0.21	37.48 ± 2.11	NS	3.73 ± 1.90 ^a^	Stable
	KPA	42.26 ± 2.11	36.89 ± 0.42	*	5.37 ± 2.53 ^a^	Stable
	CMC	40.17 ± 0.42	35.69 ± 0.42	*	4.48 ± 0.00 ^a^	Stable
	AA	38.68 ± 2.11	37.93 ± 0.21	NS	0.75 ± 2.32 ^a^	Stable

Control wine (T), wine treated with potassium polyaspartate (KPA), wine treated with sodium carboxymethyl cellulose (CMC), and wine treated with alginic acid (AA). For C_Ca_i and C_Ca_f in each wine, the mean ± standard deviation values are followed by the significance level:; ** *p* < 0.01; * *p* < 0.05; NS—not significant (according to the Student *t*-test, 5%). For ΔCa, within each wine, the mean ± standard deviation values followed by the same letter do not show statistically significant differences (ANOVA, Tukey, *p* < 0.05).

## Data Availability

The original contributions presented in the study are included in the article/Supplementary Materials, further inquiries can be directed to the corresponding author.

## Supplementary Materials

The following supporting information can be downloaded at https://www.mdpi.com/article/10.3390/foods13121880/s1: Table S1: Potassium concentration of wines (average ± standard deviation).
