# Supplementary material for: Efficiency of Alginic Acid, Sodium Carboxymethylcellulose, and Potassium Polyaspartate as Calcium Tartrate Stabilizers in Wines
Source: Foods. 2024 Jun 15;13(12):1880. doi: 10.3390/foods13121880 (PMC11202715; doi:10.3390/foods13121880)
Supplement: Supplementary file 1 [file foods-13-01880-s001.zip › foods-3042204-supplementary.pdf]

Supplementary File

Efficiency of alginic acid, sodium carboxymethylcellulose, and potassium polyaspartate as calcium tartrate stabilizers in wines

Fernanda Cosme, Luis-Filipe Ribeiro, Ana Coixão, Mário Bezerra, Fernando M. Nunes*

CQ-VR, Chemistry Research Centre, Food and Wine Chemistry Lab., University of Trás-os-Montes and Alto Douro, 5000-801 Vila Real, Portugal

* Correspondence: .fnunes@utad.pt

Table S1. Potassium concentration of wines (Average ± standard deviation)

	CKi (mg/L)
VB1	770 ± 185
VB2	609 ± 118
VB3	623 ± 80
VB4	555 ± 16
VB5	528 ± 7
VB6	454 ± 141
VB7	782 ± 36
VR1	649 ± 169
VR2	780± 21
VR3	666 ± 38
VR4	650 ± 46
VR5	764 ± 64
VT1	1075 ± 80
VT2	804 ± 98
VT3	806 ± 98
VT4	1271 ± 52
VT5	942 ± 84
VT6	1379 ± 56
VT7	1327 ± 276
